# Synthesis of sodium alginate / polyvinyl alcohol / polyethylene glycol semi-interpenetrating hydrogel as a draw agent for forward osmosis desalination

**DOI:** 10.1186/s13065-024-01246-8

**Published:** 2024-07-24

**Authors:** Taghreed Mohamed Mohamed Zewail, Menatalla Ashraf Saad, Shrouk Medhat AbdelRazik, Basma Mohamed Eldakiky, Eman Radi Sadik

**Affiliations:** 1 Chemical Engineering Department, Faculty of Engineering, Alexandria University, Alexandria, 21544 Egypt; 2 Chemical Engineering Department, Borg Al Arab Higher Institute of Engineering and Technology, Alexandria, 21933 Egypt

**Keywords:** Polyethylene glycol, Semi-interpenetrating network hydrogel, Draw agent, Forward osmosis

## Abstract

Typically, hydrogels are described as three-dimensional networks of hydrophilic polymers that are able to capture a certain mass of water within their structure. Recently, hydrogels have been widely used as drawing agents in forward osmosis (FO) desalination processes. The major aim of this study is to prepare a novel semi-interpenetrating hydrogel by crosslinking sodium alginate (SA) and polyvinyl alcohol (PVA) by using the epichlorohydrin (ECH) crosslinker and polyethylene glycol (PEG) interpenetrated within the hydrogel’s network as a linear polymer. Based on the optimum composition of SA/PVA composite hydrogel obtained from our earlier research, the effect of various percentages of PEG on the response of the hydrogel was investigated. The optimal composition of SA/PVA/PEG hydrogel was characterized by scanning electron microscopy (SEM), compression strength testing, Fourier transform infrared spectroscopy (FTIR), and X-ray diffraction (XRD). The morphological and mechanical properties of the SA/PVA/PEG semi-interpenetrating hydrogel were also compared to those of the SA/PVA composite hydrogel. Moreover, the performance of the optimal SA/PVA/PEG hydrogel in a FO batch unit as a drawing agent was investigated based on the optimal operation conditions from our previous experiments. The results showed that the optimal PEG/polymer blend mass ratio was 0.25, which increased the swelling ratio (SR) (%) of the hydrogel from 645.42 (of the neat SA/PVA hydrogel) to 2683. The SA/PVA/PEG semi-interpenetrating hydrogel was superior to the SA/PVA copolymer hydrogel in pore structure and mechanical properties. Additionally, in terms of FO desalination, the achieved water flux by SA/PVA/PEG hydrogel is higher than that accomplished by SA/PVA hydrogel.

## Introduction

The scarcity of fresh water supplies, which cannot keep up with the world’s population expansion, has been one of the most prevalent problems facing human beings in recent decades. Additionally, climate change, which produces drought in various parts of the world, made a significant contribution to this problem. Thus, by 2025, it is anticipated that nearly 60% of the population will have a drinking water shortage [1].

Nearly 97% of the water on Earth is saline water, which makes up the majority of the planet’s water. Desalination is therefore one of the most commonly used methods for creating freshwater [2, 3]. Approaches for desalination can be categorized into membrane-based and thermal approaches [4]. Nevertheless, membrane-based desalination processes are more energy-efficient than thermal desalination technologies [5]. However, while reverse osmosis (RO) is the most well-known membrane desalination technology, forward osmosis (FO) is a growing desalination method that has been recommended recently because of its higher water recovery, lower salt discharge, and lower fouling propensity. In addition, the energy consumption of seawater desalination by FO alone without the recovery of the drawing agent is less than that of RO because water molecules naturally permeate without the need for extra pressure [6, 7]. FO, which is considered a green desalination technique, transports the clean water across a semipermeable membrane that is driven by the osmotic pressure difference between the feed solution (FS) side and the draw agent (DA) side [8, 9]. The serious challenge of this technique is the choose of an appropriate DA with higher osmotic pressure and easy to be recovered [10].

There are two main classes of draw agents used in FO desalination process: organic (e.g., oligomers [11]), inorganic (e.g., ammonium carbonate solution [12]), and functionalized nanoparticles (e.g., magnetic nanoparticles [13]). In addition, DAs can be categorized into solid materials (e.g., hydrogels [14]) and liquids (e.g., ionic liquids [15]). In recent years, researchers have directed their efforts toward utilizing hydrogels as DAs for FO desalination [16]. This is owing to the decline of their toxicity levels and reverse solute flux, and their large capacity for water absorption [17]. Hydrogels are 3-D networks of hydrophilic polymeric materials that can be synthetic or natural [18]. Table 1 illustrates recent types of hydrogels used for FO desalination against distinct concentrations of the FS and their water fluxes.

**Table 1:** Recent synthesized hydrogels as draw agents for forward osmosis desalination

Hydrogel	Feed solution	J_W_ (LMH)	Reference
Bioartificial (Sodium alginate/polyvinyl alcohol) hydrogel	Distilled water	0.845 (1 h)	[27]
Green (Sodium alginate/flaxseed gum/polyethylene glycol) hydrogel	Distilled water	1.27 (1 h)	[28]
Thermo-responsive (deep eutectic mixture-co-N-isopropylacrylamide) hydrogel	DI water	2.38 (initial)	[29]
	2000ppm NaCl	1.81 (initial)	
Electro-responsive (poly(2-acrylamido-2-methyl-l-propanesulfonic acid-co-acrylamide)/polyelectrolyte polyacrylic acid) hydrogel	2 g/L NaCl	2.2 (Initial)	[30]
(polyvinyl alcohol-poly (diallyldimethylammonium chloride) hydrogel	5000 ppm NaCl	0.87 (average 6 h)	[23]
Thermo-responsive (N-isopropylacrylamide/sewage sludge ash) hydrogel	2000 ppm NaCl	2.22(average24h)	[31]
Thermo-responsive (N-isopropylacrylamide/sewage sludge ash) hydrogel	DI water	2.33(average24h)	[32]
Dual CO_2_ and thermo-responsive poly(N, N-dimethylallylamine) hydrogel	1.75% NaCl	44 (initial)	[33]

The expression of semi-interpenetrating hydrogels can be used when an existing crosslinked network is penetrated with another linear polymer without any chemical reaction. This design is applied in various aspects because of its improved swelling response and desirable mechanical properties [19]. Sodium alginate is a green polymer that is derived from brown algae [20]. When polyvinyl ester (typically polyvinyl acetate) is hydrolyzed, long-chain, water-soluble polymer known as polyvinyl alcohol (PVA) is produced. It combines the benefits of rubber and plastic while also displaying unique qualities like affordability, limited toxicity, superior mechanical behavior, and remarkable biocompatibility. Because epichlorohydrin functions as a bifunctional molecule toward hydroxyl groups in a basic solution, it was chosen as a crosslinker between SA and PVA, which are both high in OH groups [21–23]. PEG is a synthetic polymer that has been extensively studied and is FDA-approved for use in biotechnology, medication delivery, tissue engineering, and water treatment. PEG has favorable qualities such as hydrophilicity, superior mechanical characteristics, and adaptability [24–26].

This work aims to prepare a unique semi-interpenetrating hydrogel by the incorporation of PEG linear polymer in the crosslinked network of SA and PVA, and ECH was used as a crosslinker. In our prior work [27], we were able to prepare the novel SA/PVA composite hydrogel and apply it as a DA in the FO desalination process. In the present study, we wanted to improve the water uptake and mechanical performance of the former hydrogel by incorporating PEG to form a cutting-edge SA/PVA/PEG semi-interpenetrating hydrogel and apply it for the first time as a DA in the same FO unit. Depending on the optimum composition of the SA/PVA copolymer hydrogel, PVA content, and the crosslinking dose obtained from our earlier work, the influence of various percentages of PEG on the response of the hydrogel was investigated. The morphological and mechanical characteristics of SA/PVA/PEG semi-interpenetrating hydrogel were also compared to that of SA/PVA copolymer hydrogel. Moreover, the performance of the optimal SA/PVA/PEG hydrogel as a DA in a FO batch unit was investigated depending on the optimal operation conditions from our previous experiments of average hydrogel particle size and feed solution temperature.

## Materials and methods

### Materials

All the utilized materials, their specifications, and their suppliers are defined in Table 2. All of these materials were used as they were supplied, with no further purification.

**Table 2:** All chemicals used in the experiments, their purity and suppliers

Material	Specifications	Supplier
Sodium alginate (SA)	Purity (96.6%)	OXFORD LAB FINE CHEM LLP (India)
Polyvinyl alcohol (PVA)	Degree of polymerization (1700–1800 Purity (98.99%)	LOBA CHEME (India)
Polyethylene glycol (PEG)	Purity (99%)	Sigma Aldrich (USA)
Sodium hydroxide	-	Alahram company (Egypt)
Epichlorohydrin (ECH)	Purity (92%)	LOBA Cheme (India)
Acetone	Purity (99%)	ADWIC (Egypt)
Cellulose triacetate (CTA) membrane	Average pore diameter (1.21 nm) Porosity (36%) Tensile strength (33.5 MPa) Elongation (43.8%)	The national research center (Cairo, Egypt)

### Synthesis of the hydrogel

Depending on our earlier study, the optimal PVA content in the crosslinked polymer blend was 25%, and the optimum ECH/total crosslinked polymer blend mass ratio was 0.8 [27]. Thus, our present experiment was carried out as follows:

There was a solution of sodium hydroxide (8%). It was then mixed with a certain amount of SA. A specified amount of PVA was dissolved in 90 °C-heated distilled water in the meantime. Next, a homogeneous blend is achieved by combining the two solutions. To make PEG solution, distilled water is mixed with a specific mass of PEG dissolved. Afterwards, the SA and PVA polymer solution was ready, and the produced PEG solution was added. The combination was then mechanically mixed for fifteen minutes. In order to produce a homogenous paste, ECH crosslinker was next added dropwise while being continuously stirred mechanically. For the purpose of creating three samples, the above procedures were repeated using distinct PEG/total crosslinked polymer blend mass ratios of 0, 0.05, and 0.25. Maintaining the ideal composition, as previously noted, with only minor variations in PEG levels, together with maintaining a constant 8% total polymer concentration (SA and PVA) for all generated samples, is imperative. Nine hours of 75 °C curing was applied to the three produced samples. The samples of the cured hydrogel were repeatedly cleaned with heated distilled water at 60 °C until the pH approached 7. Following that, more acetone washing was performed on the samples in order to get rid of any remaining unreacted ECH. Lastly, to achieve a consistent dry weight, the cleaned samples were placed in a drying chamber set at 50 °C.

### Characterizations of the hydrogel

One of the most essential factors for evaluating hydrogels is the swelling ratio (SR). In distilled water, 0.1 g of each sample with different PEG concentration was immersed. The weight of the swollen hydrogel was measured after 1 and 24 h to determine the optimal hydrogel’s composition, which would be further characterized. A hydrogel’s swelling ratio (SR) is described as follows:

1 $$SR = \left({W_{s} - W_{d}}\right) /\: W_{d}$$

Where W_d_ is the weight of the dry hydrogel, and W_s_ is the weight of the hydrogel after swelling at a room temperature [27].

Jeol (JSM-IT200, Japan) electron scanning microscopy was used to determine the surface morphology of the optimal swollen hydrogel. After one hour of swelling, the swelled SA/PVA/PEG hydrogel sample was immediately freeze-dried under vacuum at -42 °C for three days to ensure that all absorbed water was removed. This dry sample was coated with gold for scanning.

Using the MultiTest-5xt (USA), the compressive strength of the optimal swollen hydrogel was tested at various distances. The sample was formed into a cylindrical shape. After that, it was submerged in distilled water for an hour. The swollen cylinders had the dimensions represented in Table 3. At 25 °C, the swelled samples were inserted for the compression test.

**Table 3:** Dimensions of the swollen hydrogels’ samples formed in a cylindrical shape for compressive strength test

Hydrogel	Surface area (cm^2^)	Length (cm)
SA/PVA	15.89	2
SA/PVA/PEG	7.07	2

Wide angle X-ray diffraction profiles of SA, PVA, PEG, and the optimum SA/PVA/PEG dry hydrogel powders were determined at room temperature with a X—ray powder diffractometer -XRD-D2 phaser (BRUKER, GERMANY). The 2θ range for the samples was 10–100^◦^.

Fourier transform infrared spectroscopy (Bruker Tensor 37, Germany) was utilized for the confirmation of the chemical composition of the liquid ECH and the solid SA, PVA, PEG, and the optimal SA/PVA/PEG dry hydrogel powders.

### Forward osmosis set-up

The optimized and characterized dry SA/PVA/PEG hydrogel was applied as a draw agent in a FO unit based on previously determined optimal operating conditions of average hydrogel particle size at 60 μm, FS temperature at 40 °C. The complete FO set-up was described in details in our earlier work [27]. The achieved water flux was calculated by Eq. (2).

2 $$\text{J}_\text{w}= \left(\text{W}_\text{s} -- \text{W}_\text{d}\right)\: / \:{{\rho\:}}_{\text{w}}\text{At}$$

Where J_w_ is the accomplished water flux; $\:{w}_{s}$ and $\:{w}_{d}$are the weight of the swollen hydrogel and the dried sample respectively; $\:{\:\rho}_{w}$ is the water density; A is the membrane’s surface area and t is the time of each run [34].

## Results and discussions

### Hydrogel’s characterizations

#### Swelling measurements

**Fig. 1:**
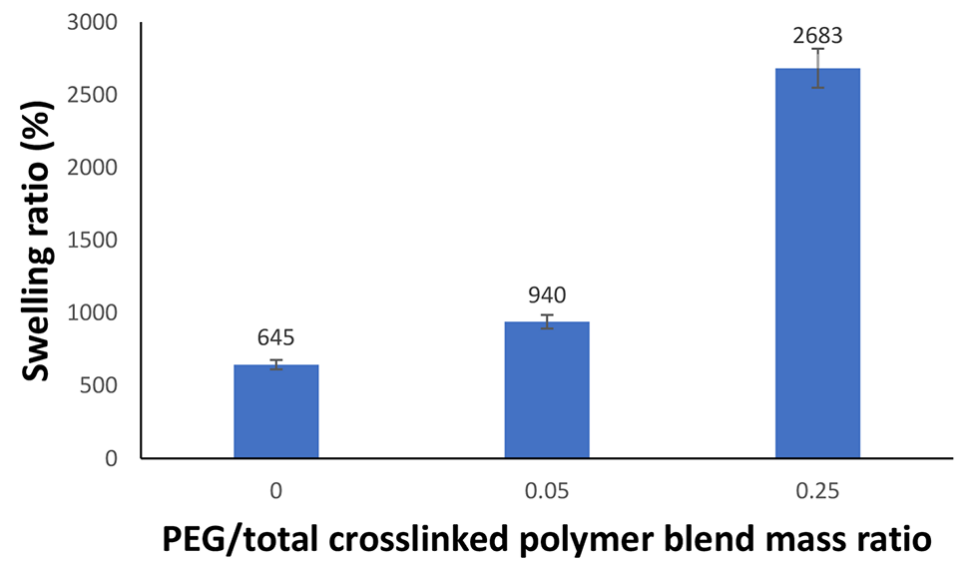
Effect of PEG incorporation on the swelling response of the hydrogel in one hour (at the optimum PVA content in the crosslinked polymer blend of 25% and ECH/crosslinked polymer blend mass ratio of 0.8)

The osmotic pressure brought on by the ionic group dissociation and the solvation force produced by the hydrogen bonding that connects the hydrogel’s network and water are what cause the polymer hydrogel to swell [23]. Figure 1 shows how the PEG incorporation impacts the swelling response of the hydrogel in an hour at the optimum PVA content and ECH dose. It is clear that there is a significant upgrade of SR (%) by raising the content of PEG. By upgrading the PEG/total crosslinked polymer blend mass ratio from 0 to 0.25, the swelling ratio (%) approximately increased to four times its value.

Two primary reasons can be used to explain these results: First off, the semi-interpenetrating network hydrogel’s structure encourages the hydrogel’s reaction to water absorption. This is carried out by enhancing water channeling within the crosslinked network [35]. The hydrophilicity of PEG, which is abundant in hydrophilic groups like O group or hydroxyl terminal group, is the second factor. These groups can create hydrogen bonds with water molecules, which improve the absorption of water [36]. There is concordance between the present trend and the preceding study [37]. It should go without saying that the optimal PEG/crosslinked polymer blend mass ratio is 0.25.

#### SEM

**Fig. 2:**
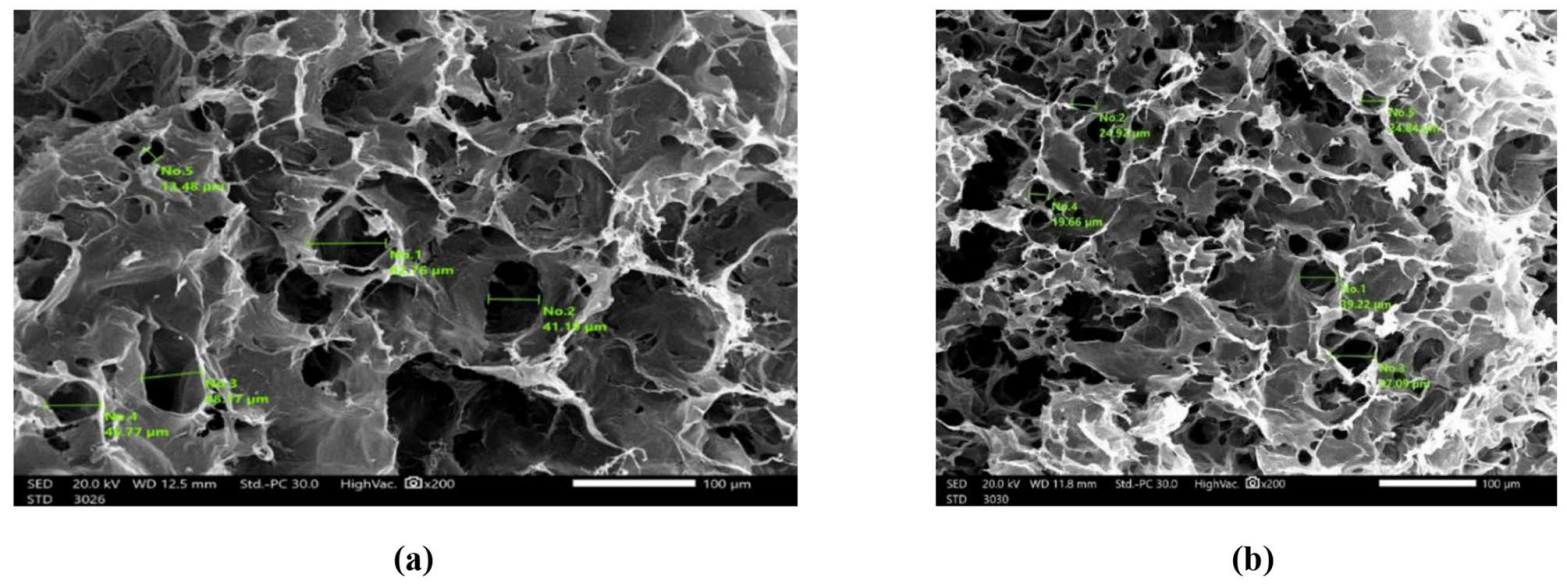
SEM (at 200× magnification) of (**a**) SA/PVA and (**b**) SA/PVA/PEG hydrogels

The porosity affects the hydrogel’s capacity to absorb water. Therefore, the morphology of the hydrogel microstructure is a crucial aspect [38]. Figure 2 depicts two separate images of the surface morphology of the optimum SA/PVA/PEG and the plain SA/PVA swollen hydrogels. Both hydrogels were examined under the same optimal PVA content and crosslinking ratio, which are mentioned above. This is done to investigate how the PEG interpenetration affects the hydrogel’s pore structure. After swelling, the hydrogel was freeze-dried, revealing pores that were originally filled with water molecules [39]. The average pore sizes of the swollen SA/PVA and SA/PVA/PEG hydrogels were 33.48 μm and 37.09 μm, respectively. It is quite astounding how the PEG’s presence enhances the hydrogel’s pore structure due to its hydrophilicity. In addition, the bulk of the hydrogel’s spherical pores are connected to one another to create an open channel system that serves as a capillary system to quickly absorb and expel water. This is because of the interpenetration of PEG as a linear polymer without any chemical reaction, which enhances capillary system creation. There is concordance between the recent trend and an older study [37].

#### Compressive strength test

**Fig. 3:**
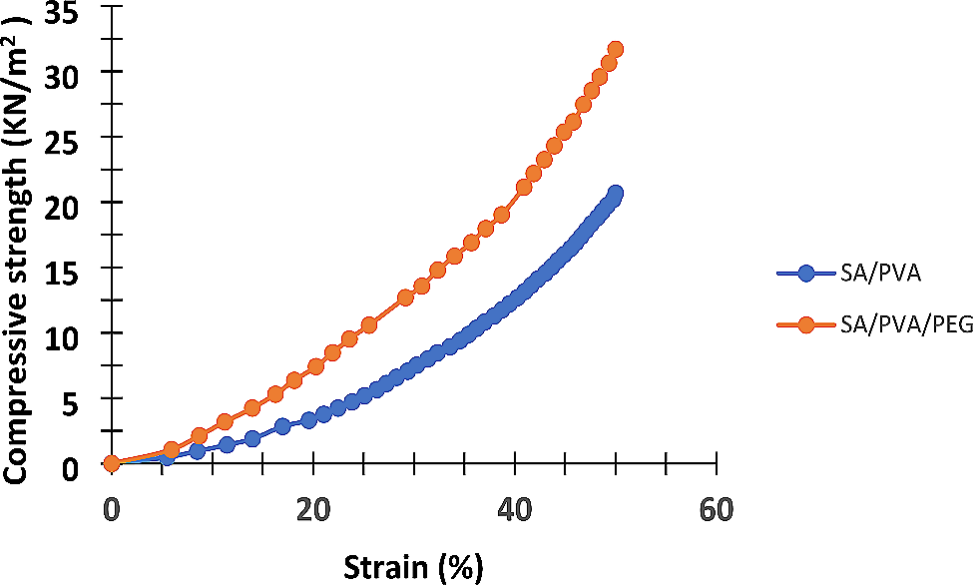
Representation of the results of the compressive strength test of SA/PVA and SA/PVA/PEG hydrogels (at 50% strain and 25 °C)

In order to illustrate how adding PEG affects the hydrogel’s mechanical strength, the neat SA/PVA and optimal SA/PVA/PEG swollen hydrogels were conducted to compression strength measurements. The samples were prepared at the same PVA content and crosslinker dose. Typically, a compression strength test is required to determine the hydrogel’s mechanical stability by regeneration through squeezing or even another way. Actually, a separate, postponed investigation may be carried out for the examination of regeneration process. The results of the compression strength test of the SA/PVA and SA/PVA/PEG hydrogels are shown in Fig. 3. When both hydrogels were compressed to their half-length, the swollen neat hydrogel could withstand a compressive strength of 20.67 KN/m^2^, while the optimal swollen SA/PVA/PEG hydrogel could tolerate a compressive strength of 31.7 KN/m^2^.

These findings demonstrate the PEG addition’s beneficial impact on the hydrogel’s mechanical strength. This is the result of three main factors. First of all, the design of semi-interpenetrating networks or even interpenetrating networks promotes mechanical properties when compared to the basic copolymer hydrogels [35, 40]. Secondly, PEG is highly regarded in many applications due to its exceptional mechanical strength [25, 41]. Thirdly and finally, with higher PEG dosages, physical entanglement and hydrogen bonding effects became more evident and served as physical crosslinking points between PEG and the polymer blend of SA and PVA. This, subsequently, requires higher compressive strength [42].

Table 4 displays the elastic modulus and compressive strength of SA/PVA and SA/PVA/PEG swollen hydrogels at a 50% strain. The term “elasticity” refers to a material’s ability to revert to its original shape and dimensions after a load is removed. It is therefore a very significant mechanical property to analyze because a hydrogel built from a polymer network uses the concept of initially developed rubber elasticity [43]. The swollen SA/PVA/PEG hydrogel had an elasticity modulus that was higher than that of the SA/PVA hydrogel. This demonstrates how using PEG improved the hydrogel’s elasticity. Actually, our findings are consistent with earlier research that looked at how PEG incorporation affected the hydrogel’s mechanical properties. In order to study how the PEG dose affects the mechanical properties of the hydrogel, Chan et al. synthesized a strong and semi-interpenetrating hydrogel from polyethylene glycol and collagen. They discovered that the upgrade of PEG concentration improved the hydrogel’s elasticity and compressive strength at 50% strain [25].

**Table 4:** The compressive strength at 50% strain and the elasticity modulus of SA/PVA and SA/PVA/PEG hydrogels

Hydrogel	Compressive strength (KN/m^2^)	Modulus of elasticity (KN/m^2^)
SA/PVA	20.67	12.19
SA/PVA/PEG	31.7	22.28

#### FTIR

**Fig. 4:**
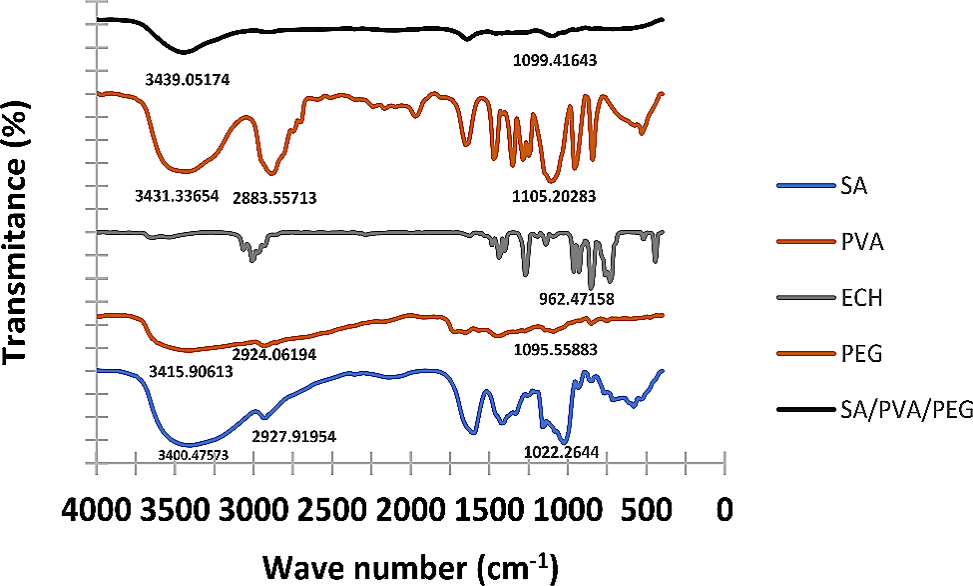
FTIR spectrum of SA, PVA, PEG, ECH, and SA/PVA/PEG

Figure 4 shows the IR spectra of SA, PVA, ECH, PEG, and SA/PVA/PEG hydrogel. The IR spectra of SA, PVA, and ECH were clearly described in our previous work [27]. For the IR spectrum of PEG, a terminal group hydroxyl is represented by the broad absorption peak at 3431.33 cm^− 1^. The peak at 2883.56 cm^− 1^ is associated with CH-stretching, while the peak at 1105.93 cm^− 1^ is connected to C-O stretching [44].

Regarding the IR spectrum of SA/PVA/PEG hydrogel, there are three main points that confirm the crosslinking reaction between SA and PVA using ECH. Firstly, the peaks corresponding to -OH stretching in SA and PVA are shifted to 3439.05 cm^− 1^. Secondly, the peak associated with acetal group stretching vanished, possibly as a result of the hydrolysis side reaction during the crosslinking reaction. Finally, the epoxide functionalities of epichlorohydrin, which are characterized by vibrations at 962 and 925 cm^− 1^, are no longer present. On the other hand, the peak at 1099.41 ensures the interpenetration of the PEG polymer chain within the network of the hydrogel without any contribution to the crosslinking reaction.

#### XRD

**Fig. 5:**
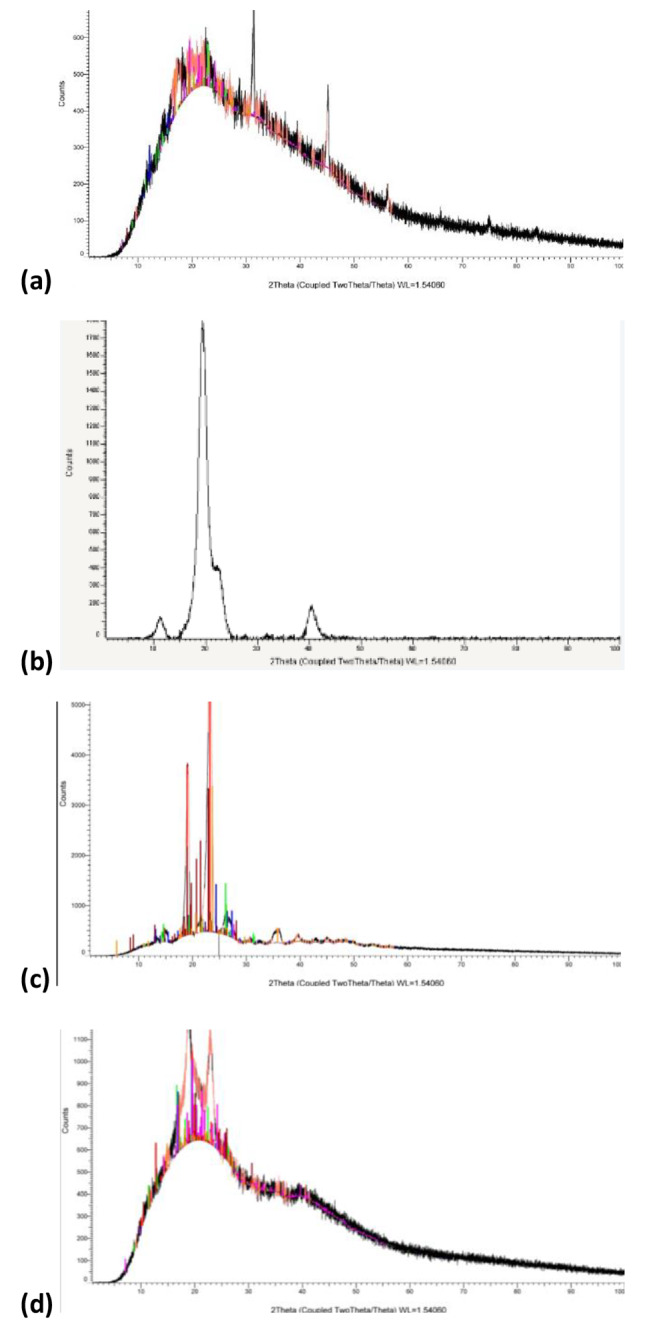
XRD spectrums of (**a**) SA, (**b**) PVA, (**c**) PEG, and (**d**) optimal SA/PVA/PEG hydrogel

Figure 5 shows the XRD of SA, PVA, PEG, and the optimal SA/PVA/PEG hydrogel. The XRD spectra of SA and PVA were well explained in our previous study [27]. PEG has been found to have a main XRD peak at 2Ɵ = 19° and 23° and other minor peaks at 26°, 36°, 39.7°, and 45° degrees, which is in agreement with findings from a prior study [45].

The SA/PVA/PEG hydrogel blend’s XRD pattern lacks any distinct peaks and exhibits a broad diffraction at 2Ɵ = 20°, which suggests an amorphous structure. The sharp and weak peaks associated with PVA and SA, respectively, have vanished, which supports the existence of crosslinks between these two substances. On the other hand, PEG is present as a linear polymer and does not interact chemically with SA and PVA, according to the hydrogel’s XRD, which nonetheless clearly shows the strong peaks at 2Ɵ = 19.31° and 23.32° that correspond to PEG.

### FO experiments

Depending on our previous study, the optimal FO operating conditions were adjusting the membrane orientation at the PRO mode (active layer facing the DA), the average hydrogel’s particle size of 60 μm, and the feed solution temperature at 40 °C. We used those optimum conditions in our current work to study the impact of PEG incorporation on the FO performance.

#### Water flux

**Fig. 6:**
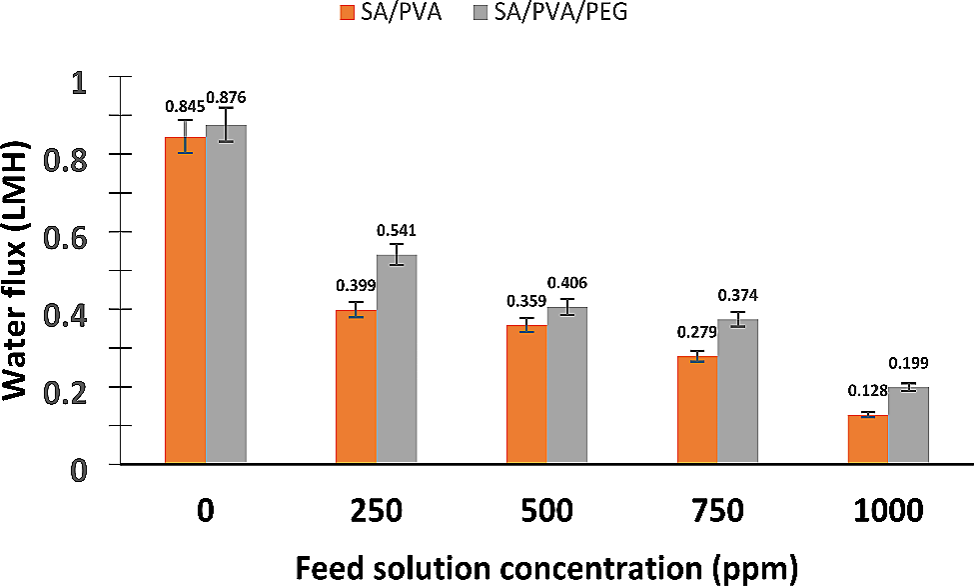
The achieved water flux by SA/PVA and the optimal SA/PVA/PEG hydrogels at different FS concentrations in 1 h (at average hydrogel’s particle size of 60 μm and the FS temperature at 40 °C)

Figure 6 represents how distinct feed solution concentrations influence the achieved water flux by SA/PVA/PEG hydrogel as DA. In addition, this figure shows a brief comparison between the performance of SA/PVA and SA/PVA/PEG hydrogels as DAs in FO desalination under the same mentioned operating conditions.

It is clear that the water flux decreases from 0.876 to 0.199 LMH as the FS concentration rises from zero to 1000 ppm. The current trend can be related to the fact that as FS’s ionic strength rises, the osmotic pressure driving force between FS and the hydrogel declines, which leads to a decreased water flux. The current findings are consistent with earlier research [37, 46], and also with our previous study on the SA/PVA copolymer hydrogel.

Moreover, it is obvious that the behavior of SA/PVA/PEG hydrogel was superior to that of SA/PVA neat hydrogel, which consequently proves the positive impact of PEG interpenetration on the water flux. This is due to the enhanced pore structure of the SA/PVA/PEG hydrogel and the hydrophilicity of the PEG polymer [35, 36]. Both reasons consequently improve the hydrogel’s capability to hold the fresh water and hence the achieved water flux.

Table 5 shows the FO performance of different semi-interpenetrating hydrogels as DAs in the FO desalination process that has been investigated in the previous studies. In addition, the behavior of the SA/PVA bioartificial hydrogel as a DA in the FO unit at different FS’s concentrations is also represented. Thermo-responsive semi-interpenetrating hydrogels have been shown to be effective as DAs in the FO desalination process by Cai et al. They produced heat-sensitive semi-IPN hydrogels by polymerizing MIPAM with poly(sodium acrylate) (PSA) or polyvinyl alcohol (PVA). Noteworthy is the fact that the FO procedure documented in this study started with hydrogels that were partially swelled and had been pre-conditioned at 40 °C. It is obvious from Table 5 that our present hydrogel outperforms the behavior of the previous semi-IPN hydrogels and also the composite SA/PVA hydrogel that was prepared and applied under the same conditions. The reasons behind that are explained in the preceding paragraph.

**Table 5:** FO performance of the present work and earlier studies

Hydrogel	Membrane	FS	Water flux (LMH)	Reference
Partially swollen (PNIPAm-IPN-PSA) (0.8 M-0.2 M)	CTA	2000 ppm NaCl solution	0.18 (1 h)	[16]
Partially swollen (PNIPAm-IPN-PVA) (0.8 M-0.2 M)			0.12 (1 h)	
Partially swollen (PNIPAm-IPN-PVA) (0.5 M-0.5 M)			0.18 (1 h)	
SA/PVA	CTA	Distilled water	0.845 (1 h)	[27]
		250 ppm NaCl solution	0.399 (1 h)	
		500 ppm NaCl solution	0.359 (1 h)	
		750 ppm NaCl solution	0.279 (1 h)	
		1000 ppm NaCl solution	0.129 (1 h)	
SA/PVA/PEG (present work)	CTA	Distilled water	0.876 (1 h)	
		250 ppm NaCl solution	0.541 (1 h)	
		500 ppm NaCl solution	0.406 (1 h)	
		750 ppm NaCl solution	0.374 (1 h)	
		1000 ppm NaCl solution	0.199 (1 h)	

#### Reverse solute flux

Due to the concentration gradient across the membrane, reverse solute flux (RSF), which has a detrimental impact on the driving force in the FO process, is defined as the solute diffusion from the draw solution side to the feed solution side [47]. Since there is no concentration gradient in the current work’s use of a hydrogel as a draw agent—a solid material with a high-water absorption capacity—reverse solute flux is minimal in this study [48]. Measurements of the conductivity of the distilled water used as the FS in the FO experiments were used to establish the RSF’s negligible value. When compared to other feed solutions with higher concentrations, distilled water provides the strongest driving force, hence conductivity tests of this solution typically provide the most accurate indicator of RSF. The distilled water’s conductivity was measured to be 0.01 S/cm both before and after the FO run, indicating that no RSF was achieved. These findings are in consistency with our previous work [27, 28].

## Conclusion

We prepared a semi-interpenetrating SA/PVA/PEG hydrogel from a crosslinked blend of SA and PVA using ECH, and PEG was interpenetrated within the hydrogel’s network as a linear polymer. The produced hydrogel was characterized using swelling measurements, SEM, compressive strength tests, FTIR, and XRD. The characterized and optimized hydrogel was applied as a draw agent in the FO desalination cell. Moreover, the performance of the currently prepared hydrogel was compared to that of the previously prepared SA/PVA copolymer hydrogel. Our conclusions were that by increasing the PEG content in the hydrogel, the swelling response of the hydrogel would be enhanced, and SA/PVA/PEG semi-interpenetrating hydrogel was superior to SA/PVA hydrogel in the pore structure and compressive strength. In addition, in the FO desalination cell, the SA/PVA/PEG semi-interpenetrating hydrogel achieved a greater water flux than the SA/PVA copolymer hydrogel. Besides, upgrading the FS concentration had a negative impact on water flux, and reverse solute flux was negligible.

## Data Availability

No datasets were generated or analysed during the current study.
